# Case Report: Alternative biopsy targets plus CEUS-guided omental puncture: enabling CLDN18.2 detection and precision therapy enrollment in pancreatic cancer

**DOI:** 10.3389/fradi.2026.1800543

**Published:** 2026-07-15

**Authors:** Hang Yin, Xiaomeng Zhang, Bing Song, Hui Li, Kun Liu, Chengzheng Zhang

**Affiliations:** 1 Jining Medical University, Jining, Shandong, China; 2 Department of Ultrasound, Affiliated Hospital of Jining Medical University, Jining, Shandong, China

**Keywords:** alternative biopsy targets, CLDN18.2, interventional ultrasound, pancreatic cancer, targeted therapy

## Abstract

**Introduction:**

Some primary pancreatic cancer lesions are located deep in the abdomen and adjacent to major blood vessels, which leads to procedural risk and low success rates of percutaneous biopsy. This situation creates substantial obstacles for pathological diagnosis and biomarker detection.

**Methods:**

We present a 54-year-old female patient diagnosed with malignant pancreatic body carcinoma. The primary tumor was in close proximity to the splenic artery and accompanied by omental metastatic nodules. After multidisciplinary team discussion, we selected the safely accessible omental metastatic lesions as alternative biopsy targets and performed core needle biopsy under contrast-enhanced ultrasound (CEUS) guidance for tissue sampling.

**Results:**

Pathological examination confirmed adenocarcinoma, and the immunophenotype was consistent with a pancreatobiliary origin. Immunohistochemical staining revealed high CLDN18.2 expression. The patient was subsequently enrolled in a clinical trial for CLDN18.2-directed combination therapy. Serial imaging follow-up demonstrated measurable shrinkage of both the primary tumor and metastatic lesions.

**Discussion:**

When imaging and clinical evaluations strongly indicate a solitary pancreatic malignancy and safely accessible metastatic lesions are present, the strategy of CEUS-guided alternative target biopsy serves as a viable approach to acquire tissue specimens and conduct biomarker testing. Nonetheless, its diagnostic concordance and clinical generalizability remain to be validated in large-sample studies.

## 1 Introduction

Pancreatic cancer is a highly aggressive gastrointestinal malignancy with an insidious onset and rapid progression. Most patients are diagnosed at an advanced stage, leading to an extremely poor prognosis (1–3). With the development of precision medicine, tumor tissue-based biomarker analysis has become an essential prerequisite for personalized treatment (4–6).

For patients whose primary tumors are located in high-risk anatomical regions where direct puncture may raise the risks of hemorrhage and sampling failure, targeting safely accessible metastatic lesions for alternative biopsy holds substantial clinical value. Nevertheless, this approach yields interpretable results only when imaging findings, clinical profiles and multidisciplinary assessments strongly support a single primary tumor origin. In addition, the histological and biomarker representativeness of metastatic lesions relative to the primary tumor requires rigorous evaluation. Herein, we present a pancreatic cancer case in which CEUS-guided biopsy of omental metastases enabled CLDN18.2 detection and subsequent precision therapy. We further elaborate on the diagnostic reasoning, technical procedures and inherent limitations of this strategy.

## 2 Case presentation

The clinical timeline for this patient is summarized in Table 1. A 54-year-old woman presented to an outside hospital in December 2024 with dull upper abdominal pain radiating to the back for more than one month. Computed tomography at that institution raised suspicion for a pancreatic space-occupying lesion. The patient had a history of elevated blood glucose, and her mother had died of pancreatic cancer. She was admitted to our hospital for further evaluation and management on January 31, 2025.

**Table 1:** Clinical timeline of the patient: key events, findings, and procedures from symptom onset to last follow-up.

Date	Event	Key findings/procedures
2024-12	Onset of dull upper abdominal pain radiating to the back	Chest CT at an outside hospital suggested a pancreatic mass
2025-01-31	Admission to our hospital	Mild cough; mild tenderness in the upper abdomen, no hepatosplenomegaly; CA19-9 46.95 U/mL, CEA 0.89 ng/mL, glucose 5.89 mmol/L; Contrast-enhanced computed tomography revealed a 32 × 25 × 27 mm mass in the pancreatic body with splenic vein invasion, multiple metastatic nodules in the perihepatic region and omentum (maximum diameter 28 mm), highly suggestive of malignancy
2025-02-06	Multimodal ultrasound evaluation and biopsy	Hypoechoic omental nodules detected on B-mode ultrasound; hypoenhancing pattern on CEUS; successful biopsy with high-quality tissue obtained under CEUS guidance, no complications
2025-02-11	Pathology and immunohistochemistry report	Omental lesion confirmed adenocarcinoma; immunohistochemistry: CK7 (+), CK19 (+), CK20 (−), CDX2 (−), WT1 (−), Ki-67 (40%), high claudin 18.2 expression (20% 2+, 70% 3+)
2025-02-27	Initiation of treatment	Enrollment in clinical trial of QLS31905 plus albumin-bound paclitaxel and gemcitabine
2025-06-11	First efficacy assessment	Primary pancreatic tumor 14 × 23 × 14 mm, largest omental metastasis 17 mm; partial response
2025-08-12	Second efficacy assessment	Primary pancreatic tumor 14 × 23 × 14 mm, largest omental metastasis 14 mm; stable disease
2025-12-03	Third efficacy assessment	Primary pancreatic tumor 13 × 23 × 14 mm, largest omental metastasis 10 mm; stable disease
Until submission	Last follow-up	No adverse events of grade 3 or higher; Eastern Cooperative Oncology Group performance status improved from 1 to 0

## 3 Imaging evaluation

Contrast-enhanced computed tomography performed on January 31, 2025 showed: ① A 32 mm × 25 mm × 27 mm mass in the pancreatic body, highly suggestive of malignancy, with mild pancreatic duct dilatation and splenic vein invasion; the lesion was closely associated with the splenic artery. ② Multiple soft-tissue nodules in the perihepatic space and peritoneum, with a maximum diameter of 28 mm, consistent with metastatic disease.

On February 6, 2025, conventional ultrasound detected a solid hypoechoic nodule in the upper abdominal omentum (Figure 1A), which had an irregular shape, relatively clear margin and heterogeneous internal echotexture. Color Doppler flow imaging (CDFI) showed a small number of punctate blood flow signals within the lesion. In accordance with the Greater Omentum Imaging-Reporting and Data System (GOI-RADS) (7), the sonographic manifestations suggested an extremely high risk of malignancy. Contrast-enhanced ultrasound (CEUS) was then performed to further define its vascular features. The examination was carried out with a GE LOGIQ E11 ultrasound system fitted with a C1-6VN convex array probe operating at a frequency of 1.5–6.5 MHz, and the mechanical index was set to 0.09. After a 2.4 mL bolus injection of SonoVue via the cubital vein, dynamic observation was conducted: the contrast agent reached the lesion at approximately 5 s and induced gradual, persistent enhancement, presenting an overall hypoenhancing pattern. Regions of interest (ROIs) were manually delineated on the solid portion of the lesion, avoiding peripheral tissues, necrotic areas and large blood vessels, and the adjacent normal tissue in the same scanning plane was selected as the control. Time-intensity curve (TIC) analysis was completed using the built-in software of the LOGIQ E11 system to obtain parameters including peak intensity (PI), time to peak (TTP) and area under the curve (AUC). The lesion displayed a global hypoenhancing pattern with a significantly decreased PI and prolonged TTP relative to surrounding normal tissues, indicating hypoperfusion and perfusion heterogeneity, which matched the typical hemodynamic features of pancreatic cancer metastases.

**Figure 1:**
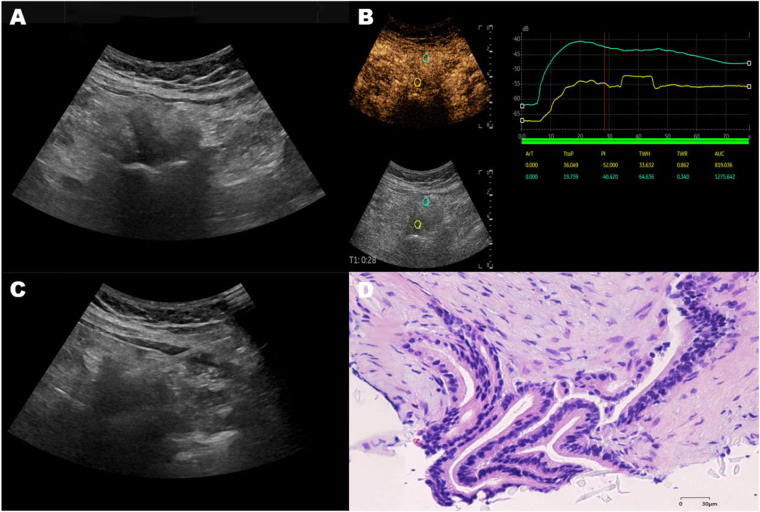
**(A)** Two-dimensional ultrasound image showing the omental nodule; **(B)** time-intensity curves derived from contrast-enhanced imaging of regions of interest (ROIs) placed on the omental lesion (yellow) and adjacent normal tissue (blue); **(C)** percutaneous needle puncture for pathological sampling of the omental nodule; **(D)** histopathological examination confirming the omental nodule as metastatic adenocarcinoma.

## 4 CEUS-guided core needle biopsy

Following MDT review, endoscopic ultrasound-guided fine needle biopsy (EUS-FNB) of the primary tumor was abandoned for two reasons: the primary lesion was tightly attached to the splenic artery, leaving no feasible EUS access route to avoid the vessel and thus carrying a high hemorrhage risk, and EUS-FNB generally retrieves a small volume of tissue that cannot meet the requirements for routine pathological examination, multiple immunohistochemistry (IHC) tests and CLDN18.2 detection. The 28-mm omental metastatic nodule, however, was superficially situated and well-demarcated from intestinal tracts and major blood vessels, making CEUS-guided puncture a safer option to obtain sufficient core tissue. Preoperative coagulation parameters were all within normal ranges. Given that peripherally enhanced regions might represent reactive hyperemia, fibrovascular stroma or areas with prominent sampling heterogeneity, we targeted the solid hypoenhancing portion of the lesion for puncture to improve the yield of representative tumor tissue. The patient was positioned supine, followed by routine disinfection and sterile draping, and received local anesthesia with 1% lidocaine. Two sampling attempts were performed using a disposable 18-gauge biopsy needle (Model: BN-1/182022-1, Nanjing Kangyou Medical Technology Co., Ltd.) with a 22-mm biopsy stroke (Figure 1C), and two intact tissue cores were harvested without coaxial technique. No hemorrhage, intestinal injury or other immediate complications were observed during 30 minutes of post-procedural ultrasound surveillance, and the patient maintained stable vital signs throughout the 24-hour routine monitoring period.

## 5 Pathological findings and clinical diagnostic rationale

Histopathologic examination of the omental nodule confirmed adenocarcinoma (Figure 1D). Immunohistochemical staining showed CK7 (+), CK19 (+), CK20 (−), CDX2 (−), WT1 (−), and Ki-67 (40%), consistent with a pancreatobiliary immunophenotype. Additional IHC staining for CLDN18.2 was performed using the EPR19202-244 antibody. Staining was interpreted according to standardized criteria, with evaluation limited to tumor cell membrane staining; cytoplasmic reactivity, non-specific background, and necrotic areas were excluded. Approximately 90% of tumor cells showed membranous staining of ≥2 + intensity, consistent with high CLDN18.2 expression.

The diagnosis was supported by integrated clinical and imaging findings. The patient had a family history of pancreatic cancer and moderately elevated serum CA19-9. Contrast-enhanced CT revealed a pancreatic body mass with mild ductal dilatation, splenic vein invasion, and close contact with the splenic artery, along with multiple peritoneal and perihepatic soft-tissue nodules consistent with peritoneal metastasis. CEUS showed hypoperfusion of the omental lesion, in line with the hypovascular nature of pancreatic adenocarcinoma. After starting CLDN18.2-targeted combination therapy, both the primary pancreatic tumor and omental metastases showed parallel and progressive shrinkage, supporting a common origin. Collectively, these findings provided consistent and convergent evidence for a pancreatic primary.

Other potential primary sites were systematically ruled out. Gastric cancer was the primary consideration in the differential diagnosis of this case. Although high CLDN18.2 expression is frequently observed in gastric cancer, this biomarker is not specific to gastric malignancy and can also be detected in certain pancreatic cancers. Routine upper gastrointestinal endoscopy was not performed in this patient, which represents a limitation of the diagnostic workup. Nevertheless, contrast-enhanced CT revealed no focal gastric wall thickening, abnormal enhancement or definite gastric mass, yielding no direct evidence to support a gastric origin. Primary ovarian cancer with pancreatic metastasis was ruled out given the absence of ovarian lesions on imaging and negative WT-1 staining. Metastatic colorectal cancer to the pancreas typically presents with positive CK20 and CDX2 expression, both of which were negative in this case, further excluding this diagnosis. Pancreatic neuroendocrine tumors are generally hypervascular lesions; however, the metastatic lesion in this patient exhibited hypoperfusion on contrast-enhanced ultrasound accompanied by markedly elevated CA19-9 levels, which were inconsistent with the typical features of pancreatic neuroendocrine tumors.

After MDT review, the final clinical diagnosis was pancreatic adenocarcinoma with omental metastasis (cT2N2M1, Stage IV). Although direct biopsy of the primary pancreatic lesion was not performed, the concordant histomorphology, immunophenotype, and imaging features provided a complete diagnostic basis to guide clinical management and subsequent targeted therapy.

## 6 Treatment and follow-up

Based on the high CLDN18.2 expression, the patient met eligibility criteria for the Phase ⅠB/Ⅱ clinical trial of QLS31905 combined with chemotherapy in CLDN18.2-positive advanced solid tumors. After providing written informed consent, she began treatment with QLS31905, nab-paclitaxel, and gemcitabine on February 27, 2025.

Results of regular imaging follow-up:

Follow-up results showed that both the primary tumor and metastatic lesions were significantly reduced after targeted therapy combined with chemotherapy (Figure 2). Serial contrast-enhanced CT imaging at four time points (January 31, 2025; June 11, 2025; August 12, 2025; and December 3, 2025) demonstrated progressive shrinkage of both the pancreatic primary lesion and the omental metastasis. The detailed imaging measurements and treatment response assessments at each follow-up time point are summarized in Table 2. The patient achieved a partial response by RECIST 1.1 criteria, followed by durable disease stability. No grade 3 or higher adverse events were observed during treatment. The patient's abdominal pain was relieved compared with baseline, and quality of life improved, confirming the efficacy of this biomarker-guided treatment regimen.

**Figure 2:**
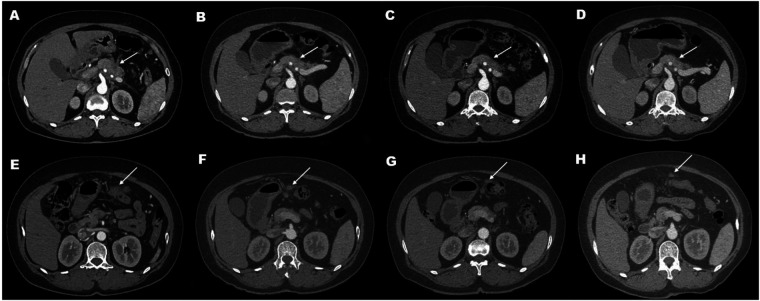
Contrast-enhanced CT findings before and after treatment. Panels **(A–D)** demonstrate the changes in the size of the pancreatic primary lesion during the arterial phase of contrast enhancement at four time points (January 31, 2025; June 11, 2025; August 12, 2025; December 3, 2025). Panels **(E–H)** demonstrate the changes in the size of the omental metastatic lesion during the arterial phase of contrast enhancement at the same four time points (January 31, 2025; June 11, 2025; August 12, 2025; December 3, 2025).

**Table 2:** Imaging follow-up of the primary pancreatic lesion and the main omental metastatic lesion: tumor dimensions and treatment response assessment at four time points.

Follow-up time	Size of the pancreatic primary lesion (mm)	Maximum diameter of the main omental metastatic lesion (mm)	Assessment
2025-01-31	32 × 25 × 27	28	Baseline
2025-06-11	14 × 23 × 14	17	Partial Response
2025-08-12	14 × 23 × 14	14	Stable Disease
2025-12-03	13 × 23 × 14	10	Stable Disease

## 7 Discussion

This single-case study explores a novel strategy for pathological diagnosis and biomarker testing by adopting alternative biopsy targets combined with contrast-enhanced ultrasound (CEUS)-guided tissue sampling when percutaneous puncture of primary pancreatic lesions carries substantial risks.

Pancreatic cancer is highly malignant and prone to early peritoneal metastasis (8). However, the primary lesion is located deep in the abdomen and adjacent to major vessels, resulting in high risk and technical difficulty for direct percutaneous biopsy. This case illustrates an innovative “alternative biopsy target” strategy: for primary abdominal tumors with high puncture risk, such as pancreatic cancer and retroperitoneal neoplasms, selecting safely accessible metastatic lesions such as omental nodules as biopsy targets represents a diagnostic strategy worthy of priority consideration. On CEUS, the omental metastatic lesion showed diffuse hypoenhancement alongside uneven perfusion across the entire lesion. These imaging findings align well with the biological features of pancreatic cancer. Its prominent desmoplastic microenvironment suppresses intratumoral angiogenesis, disrupts vascular architecture and ultimately leads to poor perfusion within tumor tissues (9, 10). CEUS can effectively distinguish viable tumor tissue from necrotic or fibrotic areas, thereby preventing non-diagnostic biopsy attempts (11, 12); it also provides real-time, dynamic guidance to direct the biopsy needle toward the most representative tumor regions, further improving the success rate of pathological evaluation and molecular testing of tissue specimens (13). A prospective study by Chen et al. (14) reported that ultrasound-guided peritoneal biopsy achieved a diagnostic success rate of 92.3% with a complication rate of only 3.7%. Arslan et al. (15) also documented the reliable diagnostic value of ultrasound-guided percutaneous biopsy for peritoneal lesions.

Pancreatic cancer remains a highly lethal digestive system malignancy. Because of the deep anatomical location of the pancreas, most patients are diagnosed at an advanced stage with poor long-term survival (16, 17). After admission, our patient underwent prompt and systematic diagnostic evaluation followed by precision therapy. Following treatment with a CLDN18.2-targeted agent combined with nab-paclitaxel and gemcitabine, the patient achieved sustained tumor regression and stable vital signs over one year, with favorable clinical outcomes. These findings validate the effectiveness of the alternative biopsy target strategy and precision therapy, leading to maximal clinical benefit for the patient. In recent years, research into CLDN18.2-targeted therapy has advanced rapidly. In patients with CLDN18.2-positive advanced gastric or gastroesophageal junction cancer, zolbetuximab plus chemotherapy showed promising efficacy in the phase Ⅲ SPOTLIGHT trial, with an objective response rate of 53.8% versus 40.2% in the control arm, median progression-free survival of 10.6 months versus 8.7 months, and median overall survival of 18.2 months versus 15.5 months, indicating superior efficacy compared with chemotherapy alone (18). In this context, the durable response observed in our patient aligns well with current progress in CLDN18.2-targeted combination therapy. While the therapeutic response observed in this patient cannot be solely attributed to the CLDN18.2-targeted agent, this case validates a practical clinical paradigm. Patients can be screened according to CLDN18.2 status determined from metastatic lesion samples and subsequently receive matched combination targeted therapy, which has yielded notable clinical benefits (19). This provides encouraging preliminary evidence to support the design of additional biomarker-driven clinical studies for refractory malignancies such as pancreatic cancer.

This is merely a single-institution case report, so it cannot validate that this strategy features universal safety, consistent reproducibility, or molecular profiles fully matching those of primary tumors; when specimens from surrogate lesions are used to guide precision therapy, clinicians must interpret outcomes comprehensively by combining imaging results, immunophenotypic data, clinical background and multidisciplinary assessments. Several key prerequisites underpin the successful use of this approach: imaging findings must strongly suggest a solitary primary abdominal tumor such as pancreatic cancer, patients must harbor at least one safely accessible peritoneal or omental metastatic nodule typically larger than 1 cm and positioned away from intestinal loops and major blood vessels, and the medical team must be skilled in multimodal ultrasound evaluation and precise biopsy procedures. As such, this technique serves as a customized solution for a specific clinical subgroup with high-risk primary lesions and accessible metastatic foci, and it cannot replace standard diagnostic pathways across all clinical settings. Future prospective multicenter studies are required to clarify the diagnostic value of surrogate biopsy strategies for various primary abdominal malignancies and determine their practical impacts on clinical treatment decisions.

## 8 Conclusion

For patients facing substantial risks from direct biopsy of primary tumors yet harboring safely reachable metastatic lesions, the CEUS-guided surrogate biopsy strategy stands as a reasonable option to obtain histological evidence and biomarker data. This case demonstrates that the technique is technically feasible under specific clinical conditions and may facilitate subsequent decision-making for precision treatment. Even so, its diagnostic concordance, scope of application and overall clinical benefits still require further verification in large-cohort studies.

## 9 Patient perspective

The patient acknowledges the professionalism of the medical team, is optimistic about her recovery, and appreciates having maintained a good quality of life.

## Data Availability

Due to the nature of this case report involving identifiable patient clinical and imaging data, the datasets cannot be made publicly available to protect patient confidentiality. Requests to access the datasets should be directed to the corresponding author and will require approval from the Ethics Committee of the Affiliated Hospital of Jining Medical University.
